# Postoperative Fillers Reduce Revision Rates in Rhinoplasty

**DOI:** 10.1093/asjof/ojad029

**Published:** 2023-03-22

**Authors:** Maria Khan, Thangasamy Sankar, Taimur Shoaib

## Abstract

**Background:**

Rhinoplasty is a complex procedure with revision rates of up to 17%. Even minor imperfections after surgery can be significant.

**Objectives:**

This review aims to investigate the use of hyaluronic acid (HA) fillers postaesthetic rhinoplasty and assess the rhinoplasty practice of the senior author.

**Methods:**

From the senior author’s practice, case records were obtained for patients who underwent surgery followed by nonsurgical rhinoplasty between 2015 and 2022. Data were retrospectively obtained and analyzed. The variables measured included the number of patients treated with fillers postoperatively, volume and type of filler used, locations of injection, and frequency of injections and complications.

**Results:**

Eight hundred patients underwent rhinoplasty between March 2015 and March 2022, and 10.6% (*n* = 85) of these underwent nonsurgical rhinoplasty using the HA filler for postoperative imperfections. The Juvederm 2 filler (Allergan, Irvine, CA) was mainly used with a mean volume of 0.2 mL. A total of 11.8% (*n* = 10) of patients had fillers for a second time and 3.5% (*n* = 3) required a third filler. The majority of patients had fillers in the rhinon area (82.3%; *n* = 70), and no complications were reported, with patient satisfaction levels being good.

**Conclusions:**

Often, there is hesitancy to use fillers after surgery due to the assumption that fillers will be required in the long term and complication rates can be high for postrhinoplasty nose fillers. From our series, we conclude that after surgery, fillers last for a greater time period than those used purely for primary augmentation. Hence, the authors recommend rhinoplasty surgeons to consider HA filler use for patients troubled by postsurgery surface irregularities.

**Level of Evidence: 5:**

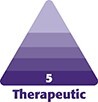

Aesthetic rhinoplasty is a surgical procedure associated with unpredictable outcomes. Surgery is usually performed with a high degree of precision and finesse, yet the best result at the end of an operation may be ruined with scar tissue causing volume or contracture imperfections.^1^ Scar tissue production is unpredictable and is preceded with inflammation and bruising, which is also usually asymmetric in nature. Scar tissue can, therefore, cause an increase in volume and can cause a contraction of tissues as well.^2^ Bone fractures will heal with callus formation, and cartilage grafts, if used, may resorb or move. Accordingly, minor imperfections following rhinoplasty are high and can cause distress and anxiety to a patient who has been concerned about the appearance of their nose for many years and is understandably hoping to have the best result possible.^2^

Revision rates following primary rhinoplasty range widely but are reported to be around 9% on average.^1^ Revisions may be for minor or major imperfections, changes that take place with time from stretching of tissues, tissue ptosis, for functional problems such as breathing difficulties, dorsal irregularities, and lack of tip definition.^3^

We have previously published our experience and technique in performing cosmetic rhinoplasty, and our average score in appearance of the nose is 4.6 out of 5.^4^ Our theory is that our revision rate is lower than the published average because we advise our patients on the need to consider fillers after surgery to smooth out postoperative imperfections. This information is listed in our Patient Information Leaflet; we inform patients about the aims of surgery prior to surgery and we record this information in our patient correspondence. We understand that patients are looking for the best possible result with minimal imperfection, and consequently, we counsel patients that sometimes such results are simply not possible without additional treatments to the nose after surgery.

We also believe that there are intervention and surgery thresholds. There is a threshold below which surgery is not indicated but patients still have a concern over their appearance despite surgery not being indicated. Such patients would normally be turned away for surgery, so we advise them about alternatives to surgery. Patients who fall below the threshold of imperfection for surgery but have concerns over the appearance of their nose postrhinoplasty may be candidates for intervention. In our practice, those patients above the threshold for intervention but below the threshold for surgery are advised to consider fillers. There is, however, no finite definition for these thresholds. Some surgeons choose to operate for minimal imperfections to try and achieve minimal changes, and some surgeons will operate only when a large change is required. Patient factors and surgeon factors are responsible for the two thresholds of intervention, these two being the threshold for fillers and the threshold for surgery. In the senior author's practice, the threshold for revision surgery is high in view of the unpredictable nature of outcomes following rhinoplasty when trying to achieve a small change.

The use of fillers in the nose is associated with complications and considerations.^5^ Many patients do not wish to have fillers after surgery as they feel fillers are temporary and surgery is permanent.^5,6^ If we could, however, inform our patients that fillers after rhinoplasty are associated with a semi-permanent outcome, then they will be less hesitant to proceed with such a strategy. Fillers in the nose are associated with complications and this organ is the second most commonly injected area associated with vascular occlusion.^6-8^ In addition, postsurgical nonsurgical rhinoplasty is associated with higher rates of complication due to the alteration of the vascular anatomy following surgery.^9,10^ We wanted to investigate complication rates following postsurgical nonsurgical rhinoplasty.

We have a theory that postrhinoplasty fillers in the nose may last longer than fillers in the nose for primary augmentation and contour smoothing. We wanted to investigate the longevity of fillers placed in the nose postrhinoplasty. We base this theory on the senior author's (T.S.) experience of one case where a patient received fillers ∼4 weeks after surgery and the resulting shape change was permanent, with the patient being discharged 3 years later with no further need for intervention.

The aim of this paper was to investigate the use of fillers postprimary aesthetic rhinoplasty, to look at areas of the nose treated, volumes, frequency, products used, and complications. Our aim was also serendipitous, to investigate and analyze the findings within the results.

## METHODS

The senior author has a practice in rhinoplasty through a clinic in London, and we looked at the case records of all patients who underwent surgical rhinoplasty followed by nonsurgical rhinoplasty, in this subset of his entire practice. All patients underwent closed rhinoplasty using the technique published by the senior author previously.^4^ We examined prospectively held data and obtained information from this data between the period from March 2015 to March 2022. We adhered to the UK Policy Framework for Health and Social Care Research guidelines.

We first extracted all data from patients who had undergone fillers. We looked at the following parameters: total number of patients treated with fillers for contour imperfections postoperatively, volume of filler used, product used, locations of injection, timelines of injections, repeated injections, the use of a needle or cannula, and complications. We looked at the total number of patients who had undergone rhinoplasty surgery. We looked to see if patients who had repeated filler treatments after rhinoplasty had their first injection later than average compared to those patients who only required one filler episode. Data were entered into a spreadsheet and simple analysis was performed using basic spreadsheet functionality.

### Treatment Method

**Figure 1.**
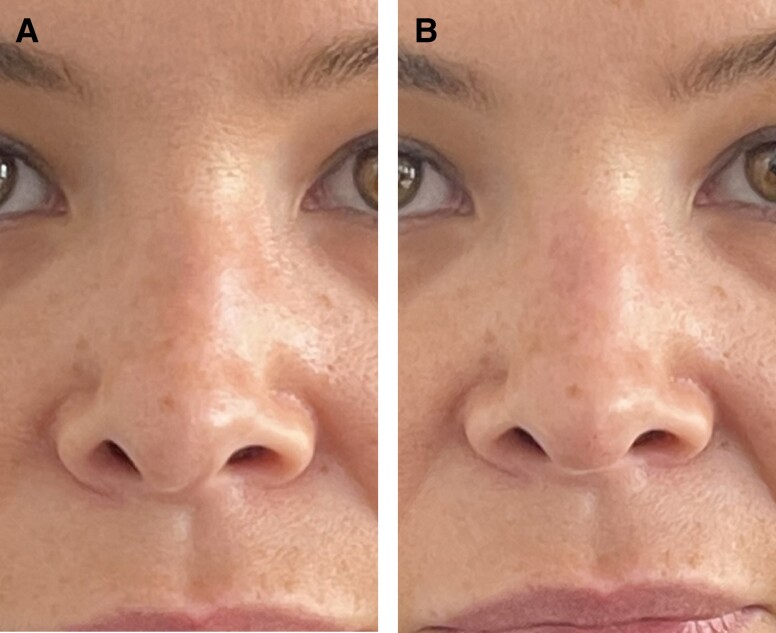
(A) Pre- and (B) post-filler treatment of a typical contour defect around the upper left sidewall of the nose. The patient was a 33-year-old female who had postoperative fillers 4 years after surgery. Juvederm 2 (0.025 mL) (Allergan, Irvine, CA) was injected into the left sidewall and 0.025 mL was injected into the left side of the columella.

**Figure 2.**
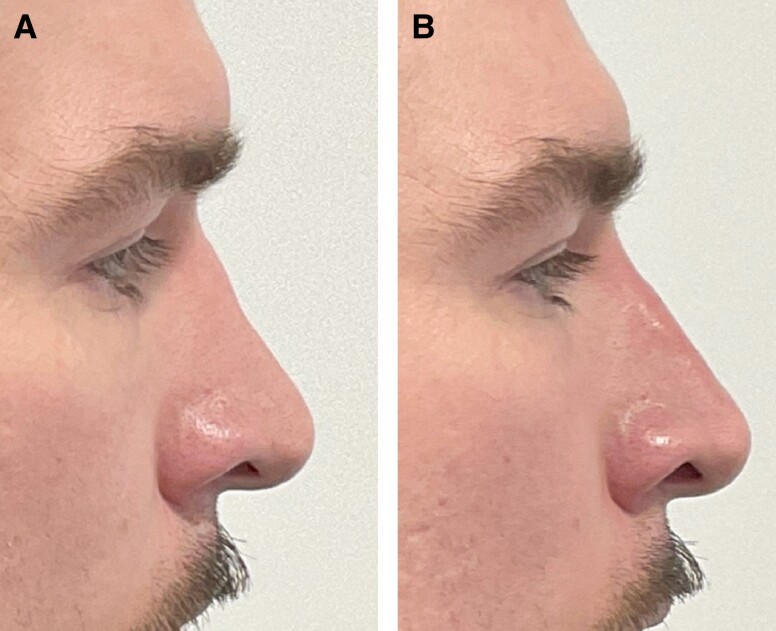
(A) Pre- and (B) post-filler treatment of contour abnormality of dosum. Patient is a 33-year-old male who had postoperative fillers 10 months after surgery. Juvederm Voluma (Allergan, Irvine, CA) was used and 0.5 mL was inserted into the dorsum.

**Figure 3.**
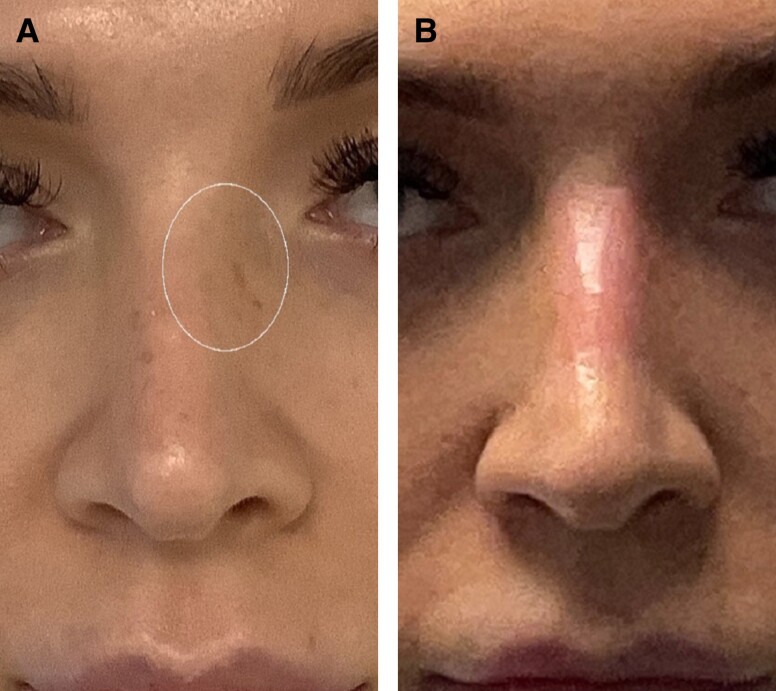
(A) Pre- and (B) post-filler treatment of convexity abnormality of side wall. Patient is a 26-year-old female who had postoperative fillers 3.5 years after surgery. Juvederm 2 (Allergan, Irvine, CA) was used and 0.05 mL was injected into the upper left sidewall of the nose.

If a patient expressed concerns over a mild contour irregularity postoperatively, the patient was counseled about their choices. Examples of the types of patient concerns that patients have concerns are shown in Figures 1-3. The choices discussed were to do nothing, to treat with fillers or to treat with surgery. The potential outcomes, aims, risks, and predictabilities of each option were discussed and if a patient chose to under fillers, we obtained written consent for the intervention. The procedure was performed in the out-patient clinic setting. The skin was sterilized with either a chlorhexidine solution or a hypochlorous solution. Simple examination gloves were used and were cleaned with rubbing alcohol solution. The needle used for injection was the one supplied with the hyaluronic acid (HA) product. A sterile gauze swab, soaked either in chlorhexidine or in hypochlorous, was held in the noninjecting hand to provide pressure to the injection point following removal of the needle postinjection. The defect requiring treatment was examined while injecting from multiple angles to ensure accurate placement of product and an optimized result. After treatment, minimal pressure was applied to the skin to reduce the risk of bruising. No pretreatment anesthetic was used to reduce the risk of skin swelling from the use of topical anesthesia. No other injections were performed during the procedure, such as vasoconstrictors. We accept that it is reasonable to use a different method for injecting, for example, decanting the product into a finer syringe and needle system, and to use local anesthetics and vasoconstrictors during the procedure, should it be the preference of the treating clinician.

## RESULTS

A total of exactly 800 patients underwent surgical rhinoplasty. Eighty-five patients had treatment with fillers after their rhinoplasty. Of these, the mean age was 29 and the age range was between 18 and 52. There were 7 males and 78 females. All patients had treatment for minor surface imperfections that fell below the threshold for surgery. The rate of patients requiring or wishing intervention after surgery was, therefore, 10.6%. In the same cohort of cases, 28 patients underwent subsequent revision surgery and the revision rate was, therefore, 3.5%. Revisions were performed for contour abnormalities, excess scarring leading to contractures and, in one case, for a subsequent alar base reduction which was deemed to be unnecessary at the time of initial surgery. The follow-up time for this study was in the range of 3 days to 1246 days (mean 330 days). The median number of filler injections in this series was 2, with a mean follow-up of 27.5 months.

In the patients who received fillers, the product used was Juvéderm Ultra 2 (Allergan, Marlow Bucks, UK) in 81 cases (95%) and Juvéderm Voluma in 4 cases (5%). On average, the volume of product used at each episode was 0.23 mL. The range of volume of product used was 0.05 to 0.6 mL during each treatment episode. In all cases, a needle was used to inject the product and a cannula was never used.

The number of times a patient underwent filler treatment ranged from 1 to 3. The mean number of episodes of injectable treatment was 1.2, with the median being one and the mode being one. In those cases where a patient had more than one treatment, the mean time gap between successive treatments was 330 days. Seventy-three patients (85.9%) had only 1 treatment, 7 patients (8.2%) had 2 treatments, and 3 patients (3.5%) 3 or more treatments.

The time between surgery and the first filler treatment episode ranged from 103 to 1246 days (3.4 years). In those patients who had 1 treatment, the mean time gap between surgery and fillers was 341 days and in those patients who had more than 1 filler treatment, the mean time gap between surgery and first filler was 350 days. There was no statistical difference between these figures when using the *t* test.

The time period for this study ranged from March 2015 to March 2022. As the practice matured, the senior surgeon realized that an increasing number of patients could undergo filler treatments. The rate of patients undergoing fillers in the first half of the study period was 31% and in the second half, it was 69%. There was no statistical difference between these figures when using the *t* test.

## DISCUSSION

In this paper, we have looked at the use of fillers postrhinoplasty to reduce the risk of revision surgery. Rhinoplasty is associated with revision rates that are higher than the senior author's rate and we believe this is because we advise patients to consider fillers for nose shape refinement postoperatively.^1^ This advice is now given to patients at the initial consultation. Some patients are engaged with such a process of using fillers and others are keen to avoid any intervention including fillers.

Our patients cite their hesitancy in using fillers after surgery, as they are under the impression that they will require fillers for life if they start putting fillers in their nose. In this study, we have shown this is not the case. In this series, the mode number of filler treatments in patients was 1.

There were no complications seen in our study. The study numbers are small (83 patients) and complication rates of nonsurgical rhinoplasty are cited as 0.35% for vascular occlusion.^11^ In this series, the average volume of product inserted was low (0.23 mL) and perhaps this is one of the reasons we have no complications to report in this study. There is no doubt, though, that vascular anatomy is altered following surgery. The nose has arterial supply through both internal and external carotid systems through vessels such as the dorsal nasal artery, the supratrochlear artery, lateral nasal artery, the angular artery, the anterior ethmoidal artery, and so on. This complexity is changed with trauma and surgery and complications are higher in the altered nose.

### Choice and Volume of Product

In this study, we used Juvéderm 2 in most cases. There are several manufacturers of fillers and within each manufacturer's product line, there are several different types of filler available. All HA fillers are different and Juvéderm 2 has its own unique properties. Juvéderm 2 is available in 0.5 mL syringes and the mean injection volume was 0.23 mL. Juvéderm 2 is relatively soft and malleable product and has very little lifting power. It is therefore ideal for postrhinoplasty defects where small volumes are required to smooth out minor surface imperfections. For primary nonsurgical rhinoplasty, other fillers are often used, such as Juvéderm Voluma, and these other fillers have greater cohesivity and therefore greater lifting and volumizing power. There is no evidence to suggest any one product is safer than another and we are aware of surgeons using fat grafting and other fillers in the nose following surgery.^1^ Given the results in this study, it seems not unreasonable to consider the use of Juvéderm 2 in the nose for minor defects or contour treatments. We chose to use Juvéderm Ultra 2 and Voluma; however, we acknowledge that there are several excellent HA fillers available for a surgeon to use. We believe it is important to state which filler was used as the two options we used are so different and yet the outcomes were so similar, that we believe it probably does not matter which fillers are used. We mainly chose to use a filler that comes in a small volume syringe. The choice of product was made through empirical data and surgeon experience. While there is no firm data available to guide surgeons on which HA filler to use for postsurgical rhinoplasty defects, the characteristics of the products were considered when choosing the filler. We recommend surgeons use products with which they are familiar. A product such as Juvéderm Ultra 2 may have a greater hydrophilic nature, and so minor defects will be smoothed out with both the effect of the filler and the effect of attracted water (much in the same way as local anesthetic injections prior to surgical rhinoplasty will mask many minor nasal contour imperfections). In those cases where a product with a higher lifting capacity and greater cohesivity was required, then Juvéderm Voluma was considered as an acceptable option. Most importantly, however, is that the surgeon is familiar with the characteristics of the filler and has knowledge of outcomes in their practice.

The volume of the product at each episode was low (mean volume used 0.23 mL) and the number of areas injected at each episode was multiple (mean number of injection points 1.94). If a cannula had been used then the number of injection points would have been impossible to count, as a cannula can cover a wide area of treatment through a single injection point. Using our technique of bolus injections with a needle, with the filler placed deep around the skeletal framework of the nose allows for targeted small-volume product placement. Juvederm 2 can be easily molded to conform to the complex irregularities that patients will sometime palpate in their nose following surgery. In our practice, the filler is placed at or around the level of the skeletal framework and this, we believe, is important for longevity of outcomes.

### Follow-up

The follow-up time for this study was in the range of 3 to 1246 days (mean 330 days). It is thought that fillers last in the nose longer than elsewhere with some reports of fillers lasting in the nose for 3 years.^1,8,12^ The reality is, though, that we do not know how long fillers will last in the nose, especially following surgery.

The median number of filler injections in this series was 2, with a mean follow-up of 27.5 months. This is low. It may be that with filler injections with a needle postrhinoplasty, the filler is injected into scar tissue which prevents the breakdown of an HA filler. Our theory is that native hyaluronidase is prevented from breaking down the injected HA, as it becomes encapsulated or encompassed within a wall of fibrosis. In other words, the theory is that filler injected into a scar will breakdown less quickly. In addition, nose filler can be injected at the level of the bone, directly onto the recently fractured periosteum. This may stimulate a periosteal reaction to create bone, or it could be that the injected HA becomes incorporated into bone callus formation. Without histological analysis of biopsied samples, we cannot be certain. It may be possible to evaluate this at some point in the future and if this becomes possible, we aim to report back with our findings. One other theory as to why the median number of filler injections is low is that patients initially find fault with their rhinoplasty result but with time they learn to live with imperfections.

As with all studies, there are limitations. In this paper, our experience is outlined as a case series. There is no control data and no comparative data with different fillers. There is no histology from patients, so we cannot tell if filler remains within scar tissue or stimulates bone production from injections, even though we speculate that these are possible reasons behind nose fillers after surgery lasting for such a long time. Future studies may wish to address these limitations.

## CONCLUSIONS

In this series, we have examined a single surgeon's practice in one geographical location. The use of fillers has increased as the practice has matured, small volumes of soft malleable filler are used, patient satisfaction remains high after filler treatment to smooth out imperfections, and surgical revision rates remain low. We would encourage rhinoplasty surgeons to consider the use of the filler in postoperative patients who have minimal surface irregularities where that contour imperfection falls below the threshold for surgery.
